# Decrease of protein phosphatase 2A subunit B by glutamate exposure in the cerebral cortex of neonatal rats

**DOI:** 10.1186/s42826-020-00064-y

**Published:** 2020-09-18

**Authors:** Ju-Bin Kang, Dong-Ju Park, Hyun-Kyoung Son, Phil-Ok Koh

**Affiliations:** Department of Anatomy, College of Veterinary Medicine, Research Institute of Life Science, Gyeongsang National University, 501 Jinju-daero, Jinju, 52828 South Korea

**Keywords:** Cerebral cortex, Glutamate, Neonate, PP2A

## Abstract

Glutamate induces neurotoxicity during brain development, causing nerve damage. Protein phosphatase 2A (PP2A) is a type of serine/threonine phosphatase that regulates various biological functions. Among the PP2A subunit types, subunit B is abundant in brain tissue and plays an essential role in the nervous system. This study investigated changes in PP2A subunit B expression through glutamate exposure in the cerebral cortex of newborn rats. Sprague-Dawley rat pups (7 days after birth) were injected intraperitoneally with vehicle or glutamate (10 mg/kg). After 4 h of drug treatment, the brain tissue was isolated and fixed for morphological study. In addition, the cerebral cortex was collected for RNA and protein works. We observed severe histopathological changes including swollen neuron and atrophied dendrite in the glutamate exposed cerebral cortex. Glutamate exposure leads to a decrease in PP2A subunit B. Reverse-transcription PCR and Western blot analyses confirmed that glutamate induces a decrease of PP2A subunit B in the cerebral cortex of newborn rats. Moreover, immunohistochemical study showed a decrease in PP2A subunit B positive cells. The reduction of PP2A subunit B expression is considered an indicator of neurodegenerative damage. These results suggest that glutamate exposure causes neuronal damage in the cerebral cortex of new born rats through a decrease in PP2A subunit B.

## Introduction

Glutamate is a excitatory neurotransmitter in the central nervous system [1]. It plays an essential role in learning and memory, synaptic plasticity, and cytoskeleton formation [2, 3]. It also contributes to regulation of synaptic development during brain development. Glutamate transporters are located in neuronal and glial cell membranes and remove glutamate from the extracellular space. However, in brain injury or disease, glutamate transporters often reverse their activity and cause accumulation of glutamate in the extracellular space. Excessive glutamate accumulation causes calcium influx into the intracellular matrix through the N-methyl-d-aspartate (NMDA) receptor channel. Calcium ion concentration is an important factor for regulation of mitochondrial function [4, 5]. High calcium ion concentration increases intracellular nitric oxide (NO) concentration and oxidative stress [6]*.* This leads to down-regulates anti-apoptosis genes and induces apoptosis cell death [7]. In addition, excessive glutamate induces excitatory toxicity and causes an ischemic cascade associated with stroke, leading to cell death and damage [8].

Protein phosphatase 2A (PP2A) is a form of serine/threonine phosphatase that is found ubiquitously in all mammalian cells. PP2A is involved in a variety of cellular functions including cell metabolism, cell proliferation, development, and apoptosis [9–11]. The PP2A structure is composed of structural subunit A, regulatory subunit B, and catalytic subunit C. Subunits A and C form dimers that bind to subunit B [12]. Subunits A and C are expressed in various tissues, but subunit B is found mainly in brain tissue. Subunit B regulates axonal growth and nervous system development [13, 14]. Therefore, PP2A subunit B is considered an important factor in maintaining neurobiological function. We have previously shown that glutamate exposure causes neonatal cerebral cortical damage by regulating various proteins [15]. We assume that glutamate exposure controls the PP2A subunit B protein during brain development, causing neuronal damage. Although the mechanism of glutamate has been reported, little information is available regarding the change in PP2A subunit B protein through glutamate exposure in the cerebral cortex of newborn animals. In this study, we investigated changes in PP2A subunit B protein during neonatal cortical damage caused by glutamate.

## Materials and methods

### Experimental animals preparation and drug administration

Sprague-Dawley rat pups were obtained from Samtako Co. (Animal Breeding Centre, Osan, Korea) and raised under consistent temperature (25 °C) and controlled light cycle (12 h light / 12 h dark) without any restriction of feed and water. During the 7 days after birth, mother rats were fed with standard chow and the pups were raised with their mother rat. Rat pups were randomly grouped as follows; vehicle- and glutamate-treated animals (*n* = 20 per group). All the experimental procedures were conducted in accordance with guidelines provided by the Institutional Animal Care and Use Committee of Gyeongsang National University (approval number: GNU-190218-R0008). Glutamate (10 mg/kg, Sigma-Aldrich, St. Louis, MO, USA) was dissolved in normal saline solution and injected into the abdominal cavity. However, only normal saline without glutamate was injected into vehicle-treated animals. After 4 h of administration, pups were euthanized by carbon dioxide gas and decapitated to separate brain tissues from skull. Cerebral cortices tissues were separated from whole brain, immediately frozen in liquid nitrogen, and preserved at − 70 °C for protein and RNA works. In addition, whole brain tissues were fixed to 4% paraformaldehyde in 0.1% phosphate-buffered saline (PBS) solution (pH 7.4) for morphological study.

### Hematoxylin and eosin staining

Brain tissue was washed with tap water to remove paraformaldehyde, dehydrated with gradient ethyl alcohols series (70 to 100%), and cleared with absolute xylene. They were embedded with paraffin using embedding center (Leica Microsystems AG, Westlar, Germany) and were solidified into paraffin blocks. Tissues were sliced with 4 μm thickness using a rotary microtome (Leica Microsystems AG) and sliced paraffin ribbons were placed on slide glass. Tissue sections were dried on slide warmer (Thermo Fisher Scientific, Waltham, MA, USA) and deparaffinized with xylene. They were then rehydrated with a series of graded ethyl alcohols (100 to 70%) and dipped in water. Rehydrated tissue sections were stained in Harris’ hematoxylin solution (Sigma-Aldrich) for 3 min and washed with tap water. Then, sections were differentiated by dipping in 70% ethyl alcohol solution containing 1% hydrochloric acid, neutralized with 1% ammonia water, and dipped in tap water. They were stained in eosin Y solution (Sigma-Aldrich) for 3 min, washed with tap water, dehydrated with a series of graded ethyl alcohols (70 to 100%), and cleared with xylene. Stained tissues were mounted with a Permount mount medium (Thermo Fisher Scientific) to permanently preserve tissue and dried on slide warmer (Thermo Fisher Scientific). They were observed under an optical microscope (Olympus, Tokyo, Japan) and histological images of the cerebral cortex were captured.

### Terminal deoxynucleotidyl transferase (TdT) dUTP nick end labeling (TUNEL) assay

TUNEL assay was carried out using ApopTag® Peroxidase In Situ Apoptosis Detection Kit (Merck Millipore, Burlington, MA, USA) to detect apoptosis. Paraffin sections were deparaffinized in xylene and dehydrated with graded ethyl alcohol series (100 to 70%). Deparaffinized sections were treated with proteinase K (20 μg/ml) for 5 min and washed with PBS for 5 min. Tissue sections were treated with 3% hydrogen peroxide in methyl alcohol for 5 min to block endogenous peroxidase. They were washed with PBS for 5 min, incubated in equilibration buffer for 1 h at 4 °C, and reacted with terminal deoxynucleotidyl transferase (TdT) labeling mixture for 90 min at 37 °C. Reaction was terminated by stop buffer for 10 min, treated with anti-digoxigenin conjugate for 1 h, and washed with PBS for 5 min. Sections were stained with 3,3′-diaminobenzidine (DAB, Sigma-Aldrich) and washed with PBS for 5 min. They were counterstained with hematoxylin, dehydrated with graded ethyl alcohol series (70 to 100%), cleaned with xylene, and coverslipped with Permount mounting medium (Thermo Fisher Scientific). Stained tissues were observed using a microscope (Olympus). Five fields of cerebral cortical images were randomly selected and TUNEL-positive cells were calculated. The apoptotic index of TUNEL assay was represented as a percentage of the number of TUNEL-positive cells to the number of total cells.

### 2-dimensional gel electrophoresis

Cerebral cortex tissues from individual animals in each groups were crushed with a lysis buffer (8 M urea, 4% CHAPS, 0.2% ampholyte, 40 mM Tris-HCl), sonicated for 3 min, and centrifuged at 20,000 g for 20 min at 4 °C. Supernatant was isolated, reacted with 10% trichloroacetic acid for 30 min in ice, and centrifuged at 20,000 g for 20 min at 4 °C. After supernatant was discarded, pellets were washed with 90% acetone, dried into vacuum, and dissolved in sample buffer [8 M urea, 4% CHAPS, 0.2% ampholyte, 40 mM Tris-HCl, 2 μg/ml dithiothreitol (DTT)]. Protein concentrations were measured using Bradford assay kit (Bio-Rad Laboratories, Inc., Hercules, CA, USA) by the manufacturer’s protocol. First-dimensional electrophoresis was performed by isoelectric focusing using immobilized pH gradient (IPG) gel strips (17 cm, pH 4–7 and pH 6–9; Bio-Rad Laboratories, Inc.). Protein sample (50 μg) was mixed with rehydration buffer (8 M urea, 2% CHAPS, 20 mM DTT, 0.5% IPG buffer, bromophenol blue) and placed on IPG gel strips for overnight at room temperature. Rehydrated IPG gel stripes were isoelectric focused using Ettan IPGphor 3 system (GE Healthcare, Uppsala, Sweden) on the following conditions: 250 V for 15 min, 10,000 V for 3 h, and 10,000 to 50,000 V. IPG strips were kept at − 20 °C for overnight and reacted with equilibration buffer (6 M urea, 30% glycerol, 2% sodium dodecyl sulfate, 50 mM Tris-HCl, bromophenol blue) containing 1% DTT (Promega Corporation, Madison, WI, USA) for 15 min, subsequently treated with equilibration buffer containing 2.5% iodoacetamide (Sigma-Aldrich) for 15 min at room temperature. Second dimensional electrophoresis was conducted on 7.5–17.5% gradient sodium dodecyl sulfate-polyacrylamide gel electrophoresis (SDS-PAGE) gel. Isoelectric focused IPG gel strips were loaded into SDS-PAGE gel and electrophoresis was performed using Protein-II XI electrophoresis equipment (Bio-Rad Laboratories, Inc.) at 10 °C with 10 mA until the bromophenol blue dye reach to the bottom of the gel. Gels were separated from electrophoresis equipment and fixed with fixing solution (12% acetic acid in 50% methanol) for 2 h. They were washed in 50% ethyl alcohol for 20 min, sensitized in 0.02% sodium thiosulfate solution for 1 min, and then washed three times in deionized water for 1 min. They were immersed dipped in silver staining solution (0.2% silver nitrate, 0.03% formaldehyde) for 20 min and washed in deionized water for 1 min. They were dipped in the development solution until the protein spots were clearly visible and reacted with a 1% acetic acid solution to stop the dyeing. The gel image was obtained using Agfar ARCUS 1200 TM (Agfar-Gevaert, Mortsel, Belgium) and the protein intensity was evaluated by PDQuest 2-DE analysis software (Bio-Rad Laboratories, Inc.). The protein spots that showed more than twice the expression intensity between the vehicle- and glutamate-treated animals were removed from the gel. The separated gel particles were decolorized with a distaining solution (30 mM potassium hexacyanoferrate, 100 mM sodium thiosulfate) and rinsed with a washing solution (10% acetic acid in 50% methanol). They were dehydrated with 50 mM ammonium bicarbonate and acetonitrile, and vacuum-dried for 20 min. Dried gel particles were reduced to a reduction solution (10 mM DTT in 0.1 M ammonium bicarbonate) at 56 °C for 45 min, dehydrated again with 0.1 M ammonium bicarbonate and acetonitrile, and vacuum-dried for 20 min. They were reacted with a digestion solution (12.5 ng/ml trypsin, 0.1% octyl beta-D glycopyranside in 50 mM ammonium bicarbonate) for overnight at 37 °C. The digested protein was extracted by extraction buffer (1% trifluoroacetic acid in 66% acetonitrile) and vacuum-dried for 2 h. The dried proteins were prepared by mixing the extraction buffer and the matrix solution (alphacyano-4-hydroxycinnamic acid and nitrocellulose of acetone) and placed on the matrix-assisted laser desorption ionization-time (MALDI-TOF) metal plate (Aurora Biomed Inc., Vancouver, Canada). MALDI-TOF was performed using Voyager-DE STR (Applied Biosystem, Foster City, CA, USA) and peak results were obtained. The peak results were analyzed through NCBI and MS-FIT protein sequence databases and a list of protein data was recorded.

### Reverse transcription polymerase chain reaction

Total RNA was extracted using Trizol Reagent (Thermo Fisher Scientific**)** by the manufacturer’s manual. Superscript III first-strand system **(**Thermo Fisher Scientific**)** was used to r**e**verse transcription process according to the manufacturer’s instructions. Complement DNA (cDNA) as a template was synthesized from total RNA (1 μg). Polymerase chain reaction (PCR) was performed under the following conditions using the primer corresponding to the cDNA: an initial step 5 min at 94 °C; 5 min at 94 °C; 30 s at 94 °C, 30 s at 54 °C, and 1 min at 72 °C for 30 cycles; and a final extension step for 10 min at 72 °C. The following primers were used: PP2A subunit B (223 bp), forward primer: 5′-CCTGGTATGCCAAACTCGAT-3′, reverse primer: 5′- ACAATAGCCACCTGGTCGTC-3′; β-actin (238 bp), forward primer: 5′-GGGTCAGAAGGACTCCTACG-3′, reverse primer: 5′-GGTCTCAAACATGATCTGGG. The amplified products were mixed with Loading STAR dye (Dyne Bio, Seongnam, Korea) and loaded into 1% agarose gel. The electrophoresis was performed using Mupid-2 plus (Takara bio, Shiga, Japan). The products were visualized under ultraviolet light and the images were obtained using a Gel documentation systems (Bio-Rad Laboratories, Inc.).

### Western blot analysis

Cerebral cortices were homogenized in lysis buffer (1 M Tris-HCI, 5 M sodium chloride, 0.5% sodium deoxycholate, 10% sodium dodecyl sulfate, 1% sodium azide, 10% NP-40) with phenylmethylsulfonyl fluoride (200 μM), which inhibits protease. Homogenates were kept in ice for 1 h, sonicated for 3 min, and centrifuged at 15,000 g for 20 min at 4 °C. Supernatants were isolated from centrifuged samples and remained pellets were discarded. Protein concentration was measured using bicinconinic acid kit (Thermo Fisher Scientific) by the manufacturer protocol. Total protein (30 μg) from each samples were loaded on a 10% SDS-PAGE gels and electrophoresed at 10 mA for 30 min using Mini PROTEAN Tetra Cell (Bio-Rad Laboratories, Inc.). Electrophoresed proteins were transferred to poly-vinylidene fluoride (PVDF) membrane (Millipore, Billerica, MA, USA) using Mini Trans-Blot Cell (Bio-Rad Laboratories, Inc.) at 120 V for 2 h. Transferred membranes were incubated with 5% skim milk in Tris-buffered saline containing 0.1% Tween-20 (TBST) for 1 h to prevent non-specific binding. Membranes were washed with TBST and incubated with primary antibody for overnight at 4 °C. Anti-PP2A subunit B antibody (diluted 1:1000, rabbit IgG, Cell Signaling Technology, Beverly, MA, USA) and anti-β-actin (diluted 1:1000, mouse IgG, Santa Cruz Biotechnology, No. SC-47778) were used as primary antibodies. After primary antibody reaction, membranes were washed with TBST and incubated with horseradish peroxidase-conjugated secondary antibody (1:5000, anti-mouse IgG or anti-rabbit IgG, Cell Signaling Technology) for 2 h at room temperature. Membranes were washed with TBST and incubated with enhanced chemiluminescence (ECL, GE healthcare). They were exposed on X-ray films (Agfar-Gevaert) by the manufacturer’s protocol. Optical density was evaluated using Sigmagel 1.0. (Jandel Scientific, San Rafael, CA, USA) and SigmaPlot 4.0 (SPSS Inc., Point Richmond, CA, USA).

### Immunohistochemical staining

Brain tissues were fixed with 4% neutral buffered paraformaldehyde in PBS (pH 7.4) for 24 h. They were washed with tap water for overnight, dehydrated through a series of graded ethyl alcohol (70 to 100% ethyl alcohol), immersed in xylene, and embedded with paraffin. Brain tissues were cut to 4 μm thick and paraffin ribbons were placed on gelatin-coated slides. These slides were immersed in xylene, rehydrated through a 100 to 70% graded ethyl alcohol, and soaked in deionized water. Tissue sections were microwaved in 10 mM sodium citrate buffer (pH 6.0) for 10 min as a heat induced epitope retrieval. They were cooled at room temperature for 1 h and washed 3 times for 10 min in PBS. They were immersed in 1% hydrogen peroxide in methyl alcohol for 10 min as an endogenous peroxidase block step. They were washed 3 times for 10 min in PBS and blocked with non-specific protein by applying with 1% normal goat serum for 1 h. Tissue sections were applied with anti-PP2A subunit B antibody (diluted in 1∶100, Cell Signaling Technology) in humidified incubator for overnight at 4 °C and washed with PBS. They were reacted with biotinylated goat anti-rabbit secondary antibody (diluted in 1:200, Vector Laboratories, Burlingame, CA, USA) at room temperature for 2 h, washed with PBS, and incubated with avidin-biotin-peroxidase complex (Vector Laboratories). They were washed with PBS and applied with DAB solution containing 0.03% hydrogen peroxidase. They were rinsed with PBS, counterstained by hematoxylin, and washed with tap water. They were dehydrated with gradient ethyl alcohol series (70 to 100%) and immersed in xylene. Tissue sections were mounted by mounting media (Thermo Fischer Scientific) and observed by Olympus optical microscope (Olympus). Cerebral cortex images were taken in four random areas (500 × 500 μm) from each animal. The number of total cells and PP2A subunit B positive cells was counted in selected area. Expression of PP2A subunit B positive cells was calculated by following formula: 100 × (number of PP2A subunit B positive cells / number of total cells).

### Statistical analysis

All results data are average ± standard error of mean (S.E.M.). Statistical analyses were conducted using SigmaGel 1.0 (Jandel Scientific, San Rafael, CA, USA) and SigmaPlot 4.0 (SPSS Inc., Point Richmond, CA, USA). The data were compared with one-way analysis of variance (ANOVA) and then Student’s *t-*test was conducted. When the *p* value was lower than 0.05, the results were considered to be significantly different.

## Results

**Fig. 1:**
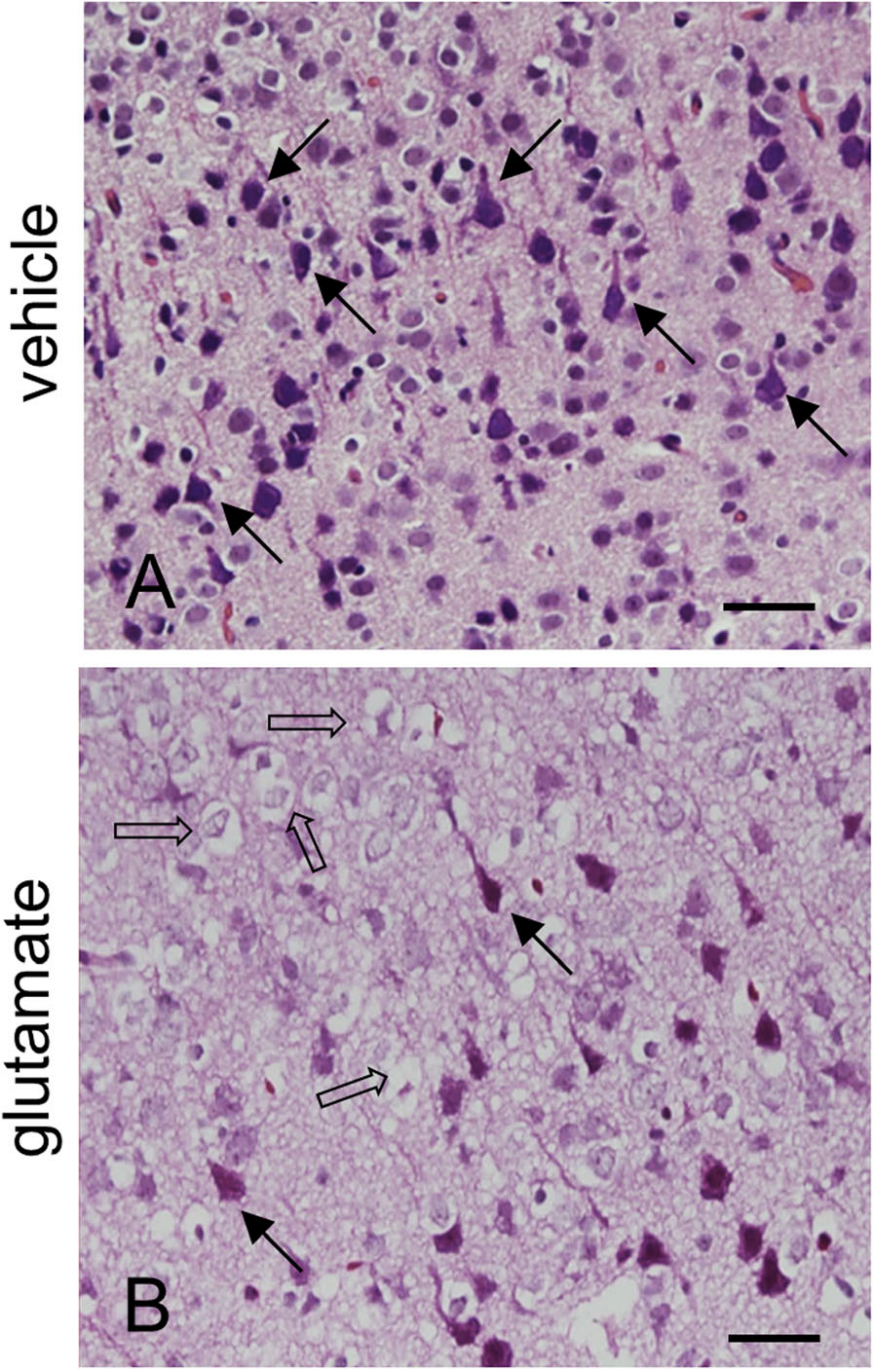
Representative photomicrographs of hematoxylin and eosin staining in neonatal cerebral cortices of vehicle- (**a**) and glutamate-treated animals (**b**). Closed arrows indicate normal neurons with the typical pyramidal shape and developed dendrites. Open arrows indicate damaged neurons with the atypical round shape and swelled nucleus. Scale bar: 100 μm

**Fig. 2:**
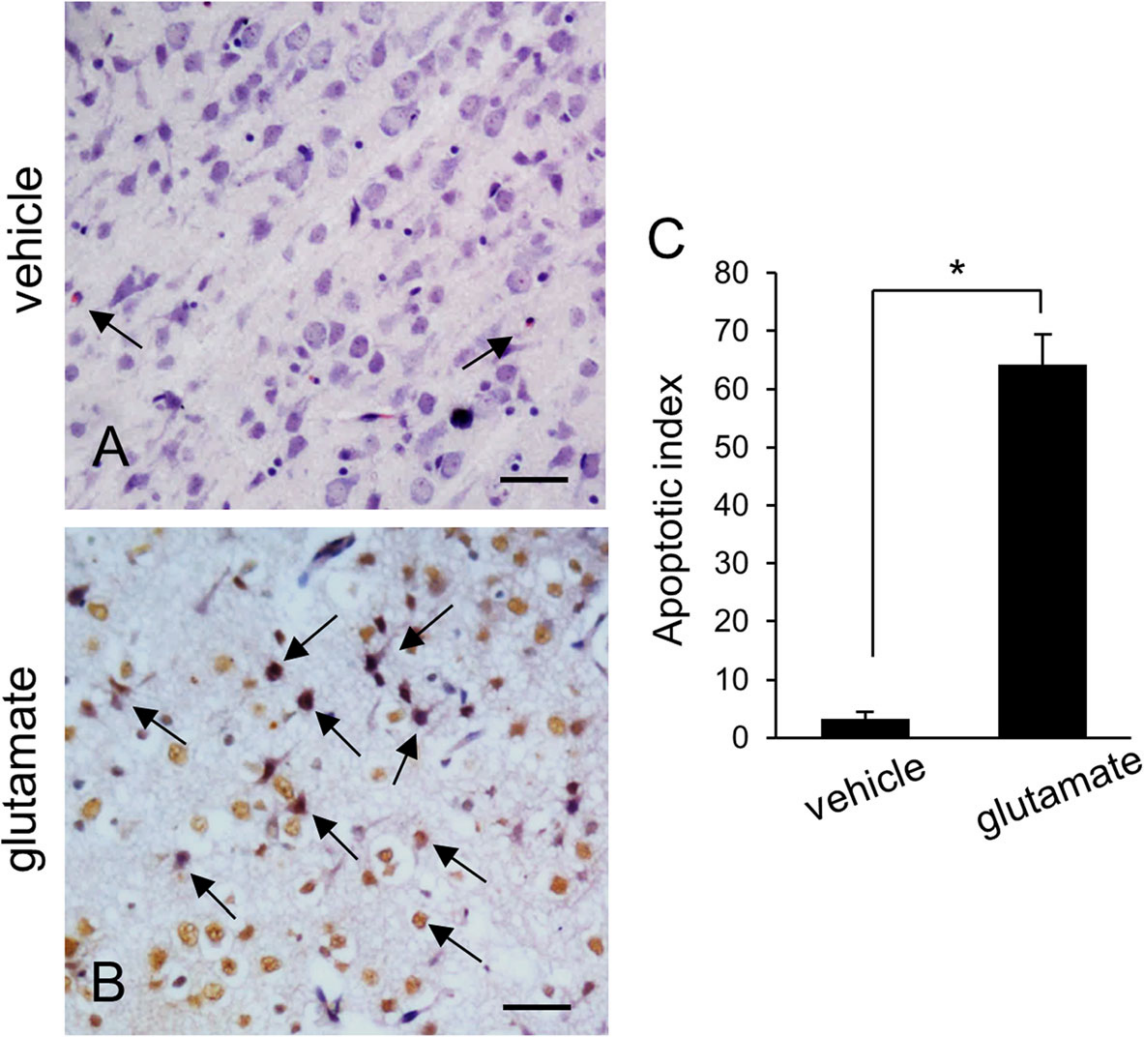
Representative photos of TUNEL staining and apoptotic index in neonatal cerebral cortices of vehicle- (**a**) and glutamate-treated animals (**b**). The number of TUNEL positive cells was increased in glutamate-treated animals. Arrows indicate TUNEL-positive cells. Scale bar: 100 μm. Apoptotic index indicates the percentage of the number of TUNEL-positive cells to the number of total cells (**c**). Data (*n* = 5) are shown as mean ± S.E.M. * *P* < 0.05

**Fig. 3:**
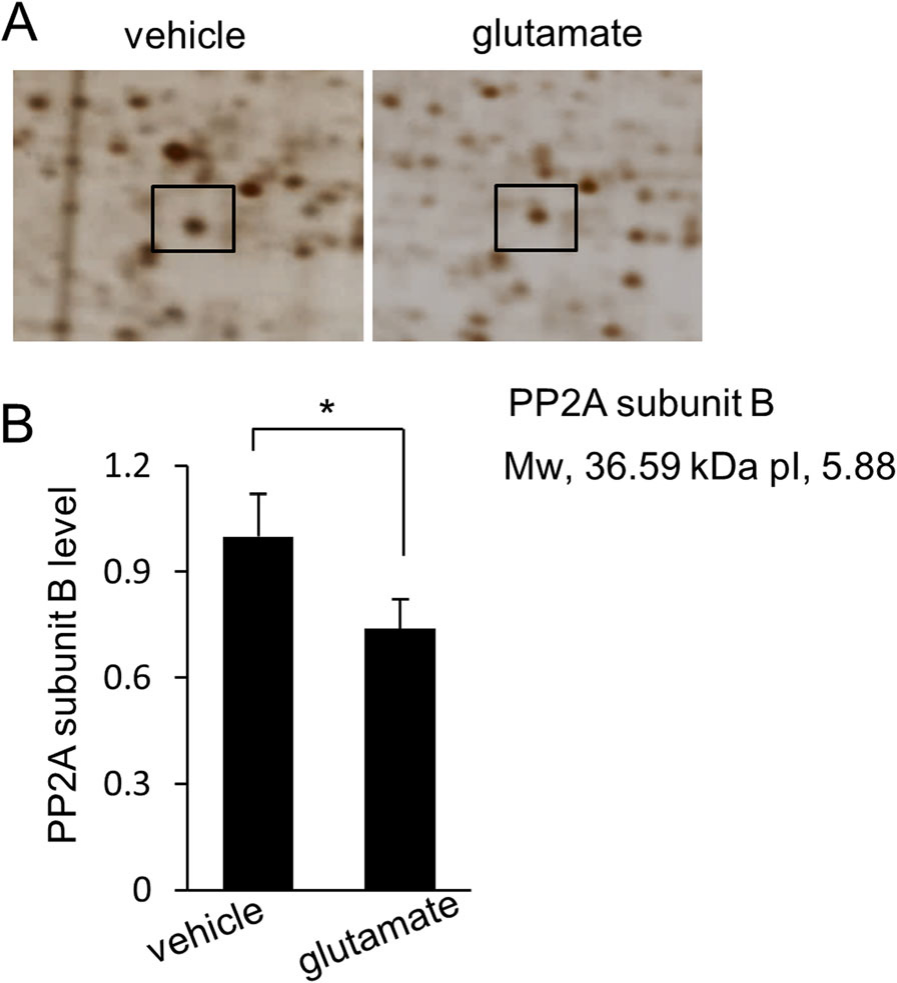
Image of protein phosphatase 2A (PP2A) subunit B protein spots in neonatal cerebral cortices of vehicle- and glutamate-treated animals (**a**). Squares indicate (PP2A) subunit B protein spots. The intensity of the spots was measured using PDQuest software. The ratio of intensity is described as spot intensity of glutamate-treated animals to spots intensity of vehicle-treated animals (**a**). Data (*n* = 5) are shown as mean ± S.E.M. *P < 0.05

**Fig. 4:**
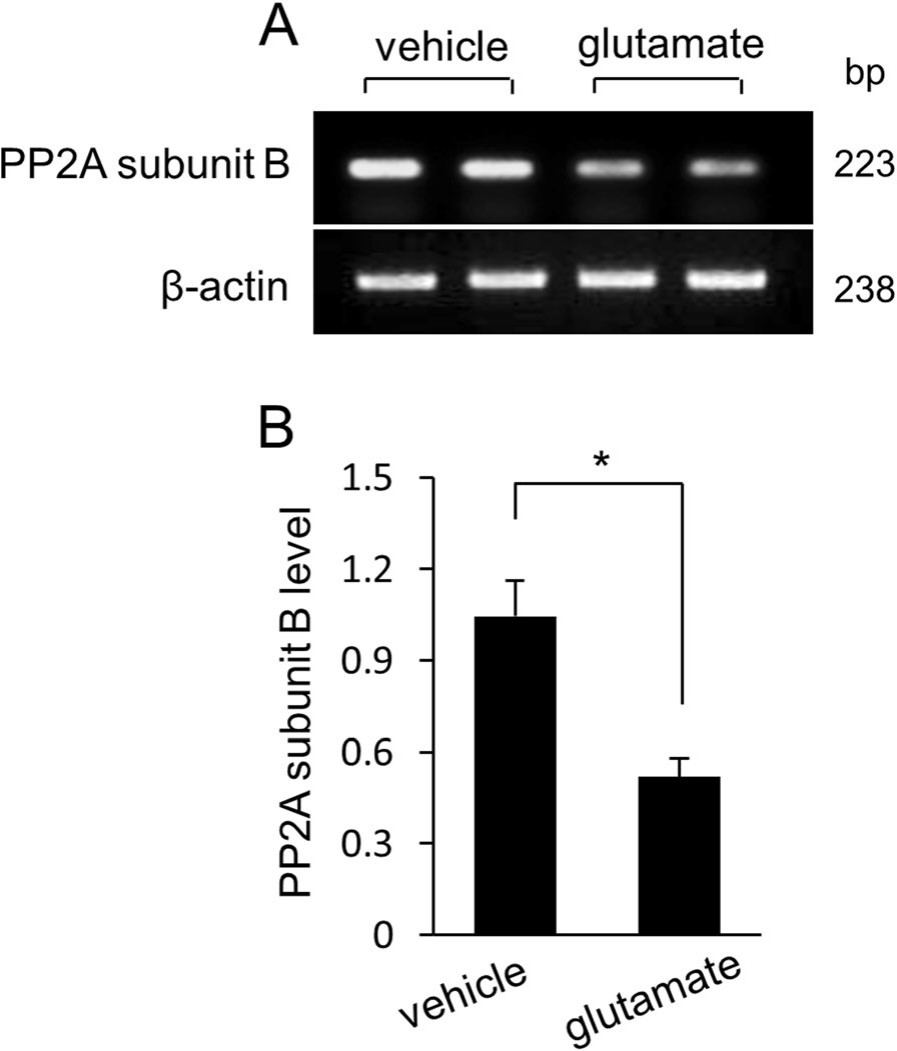
Reverse transverse-PCR analysis of protein phosphatase 2A (PP2A) subunit B in neonatal cerebral cortices of vehicle- and glutamate-treated animals (**a**). Each lane represents an individual experimental animal. Densitometric analysis is represented as intensity of PP2A subunit B to intensity of β-actin (**b**). Data (*n* = 5) are represented as mean ± S.E.M. *P < 0.05

**Fig. 5:**
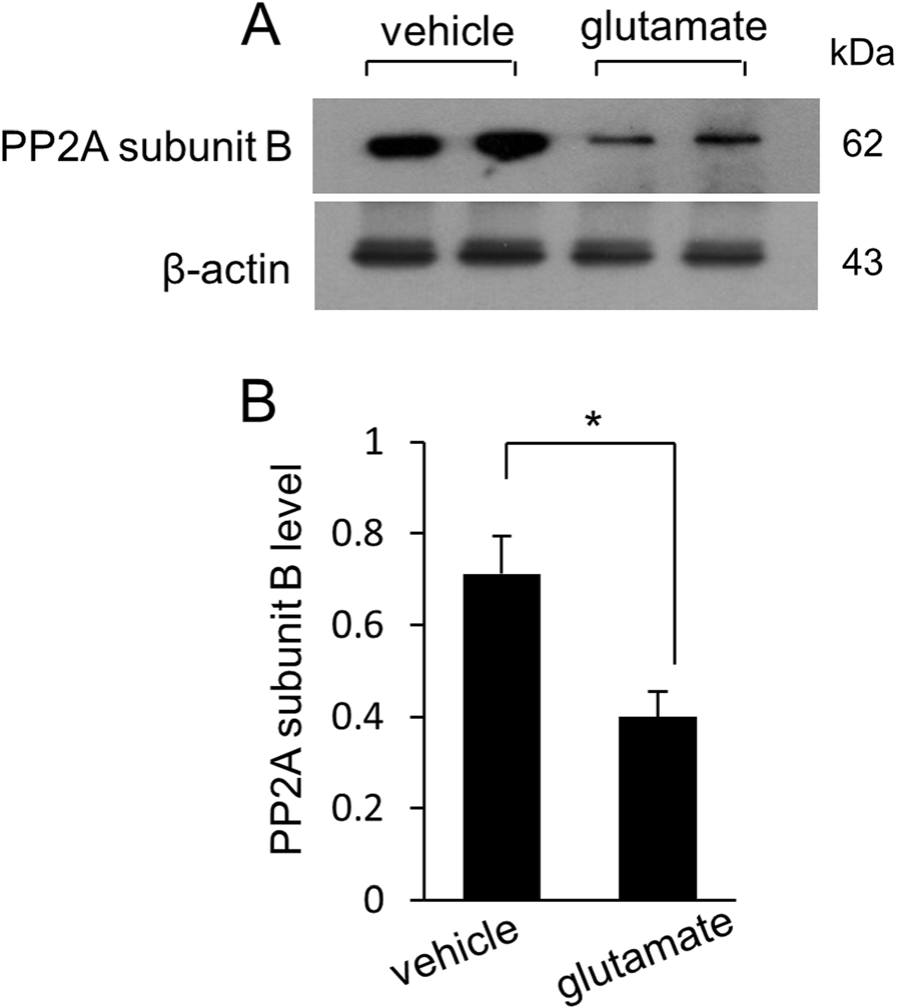
Western blot analysis of protein phosphatase 2A (PP2A) subunit B in neonatal cerebral cortices of vehicle- and glutamate-treated animals (**a**). Each lane represents an individual experimental animal. Densitometric analysis is represented as intensity of PP2A subunit B to intensity of β-actin (**b**). Data (*n* = 5) are represented as mean ± S.E.M. *P < 0.05

**Fig. 6:**
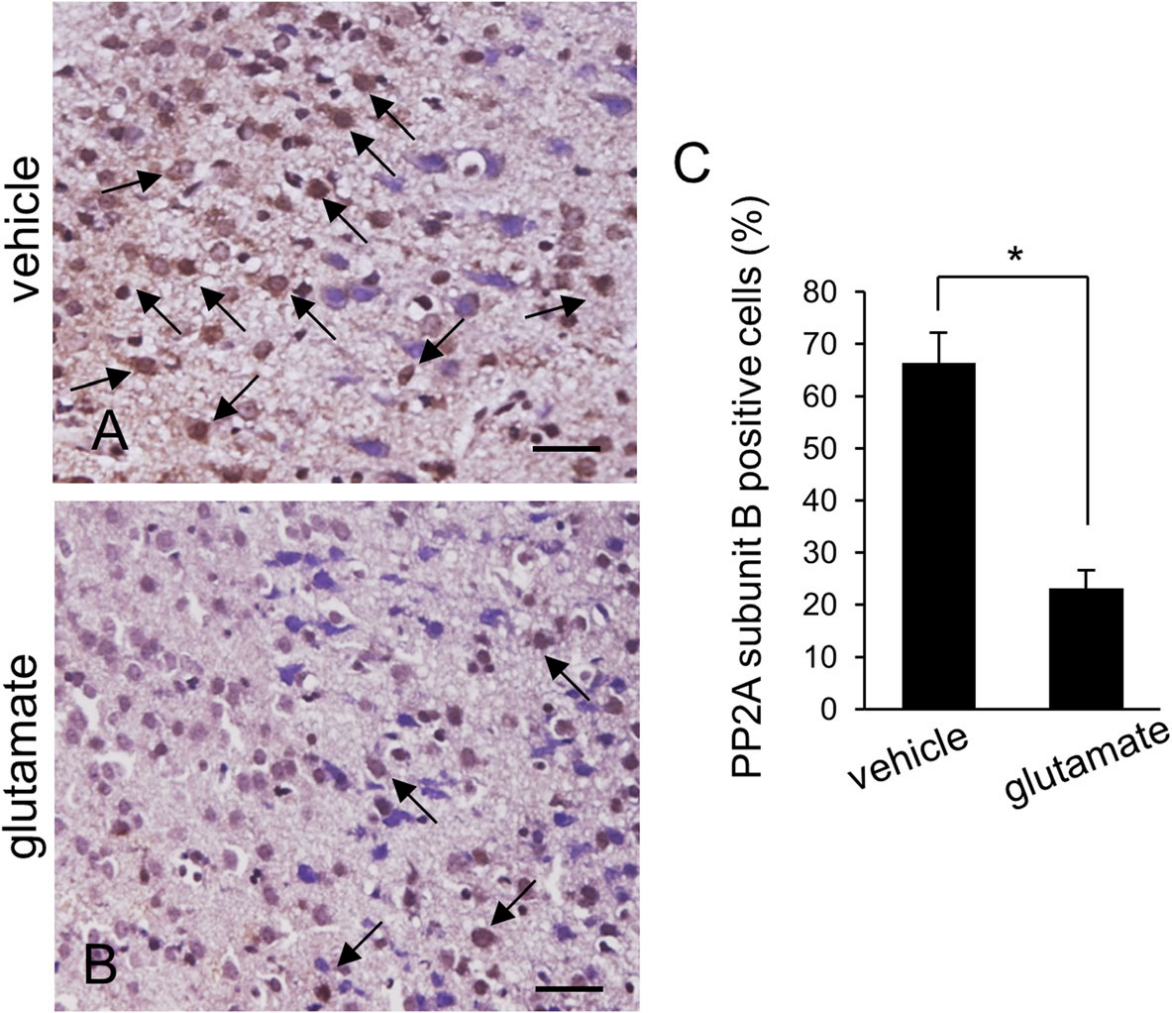
Immunohistochemical staining photographs of protein phosphatase 2A (PP2A) subunit B in neonatal cerebral cortices of vehicle- (**a**) and glutamate-treated animals (**b**). Arrows indicate positive cells of PP2A subunit B. PP2A subunit B positive cells expression levels was decreased in glutamate-treated animals (**c**). Scale bar = 100 μm. Data (*n* = 5) are shown as mean ± S.E.M. * *P* < 0.05

We confirmed that glutamate exposure causes severe histopathological changes in the cerebral cortex of newborns rats. Normal neurons in a pyramidal shape were observed in vehicle-treated animals. These neurons contained a cell body with round and large nucleus, and well-stretched dendrites. However, swelled neurons with round cell bodies and atrophied dendrites were observed in glutamate-treated animals (Fig. 1). TUNEL-positive cells were observed as marker for neuronal damage. TUNEL-positive reactions were remarkably appeared in glutamate-treated animals. Vehicle-treated animals contained a few TUNEL-positive cells. However, the number of TUNEL positive cells was significantly increased in the glutamate-treated animal. (Fig. 2a, b). The apoptotic index represent as the value of TUNEL-positive reactions. They were 3.28 ± 1.25% and 64.15 ± 3.27% in vehicle- and glutamate-treated animals, respectively (Fig. 2c). Figure 3 shows a decrease in PP2A subunit B expression by glutamate exposure in the cerebral cortex of newborn rats using proteomic analysis. The peptide mass of the PP2A subunit B is 9/56 and the sequence is 29%. The proteins intensity of the vehicle-treated animal was set to 1 to evaluate the change in glutamate-treated animal. The relative expression level of PP2A subunit B was 0.73 ± 0.07 in the glutamate-treated animal. In addition, reverse transcription PCR and Western blot analyses showed a decrease in PP2A subunit B expression level due to glutamate exposure. The level of PP2A subunit B expression was evaluated by the intensity ratio of β-actin. The respective transcript levels of PP2A subunit B were 1.05 ± 0.15 and 0.52 ± 0.03 in vehicle- and glutamate-treated animals (Fig. 4). Relative PP2A subunit B protein levels were 0.71 ± 0.08 and 0.40 ± 0.05 in vehicle- and glutamate-treated animal, respectively (Fig. 5). In addition, the results of immunohistochemical staining showed a decrease in PP2A subunit B expression by glutamate exposure. Positive cells of PP2A subunit B were detected in pyramidal neurons in the cerebral cortex of newborn rats. The number of PP2A subunit B-positive cells was decreased in glutamate-treated animals compared to vehicle-treated animals. PP2A subunit B positive cells expression levels were 66.42 ± 5.78% and 23.15 ± 3.51% in vehicle- and glutamate-treated animals, respectively (Fig. 6).

## Discussion

Glutamate is the most abundant excitatory neurotransmitter in the nervous system. It plays an essential role in regulating growth cones and synapses during brain development. It is also involved in cognitive functions such as brain learning and memory [16]. However, overexposure of glutamate causes oxidative stress and neuronal cell death [17]. We have previously shown that glutamate treatment induces neonatal cerebral cortical damage through modulation of various proteins [18]*.* This study showed a decrease in PP2A subunit B expression during glutamate-induced neonatal brain cortical injury.

PP2A subunit B acts as an essential component for the performance of neurobiological functions. PP2A is an important enzyme that regulates various cellular activities such as cell metabolism, cell division, and cell death [9–11]. PP2A subunit B regulates axonal growth and neuronal development [10, 19]. We previously confirmed a decrease in PP2A subunit B in the cerebral cortex of a stroke animal model [18]. In addition, we reported that various reagents including ferulic acid and quercetin exert neuroprotective effect against ischemic brain injury through PP2A subunit B expression [20, 21]. We clearly showed change of PP2A subunit B by those reagents in vitro and vivo models of cerebral ischemia. PP2A subunit B is an important factor and also can be an indicator in maintaining neurobiological function. Therefore, reduction of PP2A subunit B is considered one of the many causes of nervous system diseases. In this study, we identified a decrease in PP2A subunit B protein by glutamate exposure in the newborn cortex through a proteomic study. Reverse transcription PCR and Western blot analysis confirmed that glutamate significantly reduces PP2A subunit B level. Dysregulation of PP2A is associated with neurodegenerative diseases including Parkinson’s disease and dementia [22]. Moreover, a decrease in PP2A has been observed in Alzheimer’s disease [23]. Decreasing PP2A activity induces intracellular neurofibrillary entanglement and leads to neurodegenerative disorders [24]. PP2A is related to the tau protein. Tau protein exerts abundantly in neurons in the central nervous system, maintains microtubule stability in axons, and promotes tubulin polymerization. Hyperphosphorylated tau aggregates neurofibrillary tangles and causes central nervous system disorders [24]. In clinical practice, neurodegenerative diseases such as Alzheimer’s disease and Parkinson’s disease have been associated with superphosphate tau [24, 25]. PP2A dephosphorylates tau proteins and stabilizes the nervous system [26]. Our results indicate that a decrease of PP2A subunit B protein by glutamate exposure induces phosphorylation of tau and results in neonatal cerebral cortical neuron damage. Glutamate toxicity causes oxidative stress and neuronal cell death. Oxidative stress inhibits oxidation reduction homeostasis and consequently causes cell death. We have previously shown that glutamate exposure induces ischemia and results in neuronal cell death in cultured neurons [27, 28]. In addition, a decrease of PP2A subunit B was confirmed in cultured adult neurons [18]. A balance between protein kinase and phosphatase activity is required for normal cellular functions. Therefore, phosphatase is strictly regulated as a kinase. A decreased PP2A activity by glutamate exposure during neuronal development interferes with the balance of protein kinase and phosphatase activity causing neurodegenerative disorders. We have demonstrated histopathological changes caused by glutamate exposure in the neonatal cerebral cortex. We also confirmed that glutamate induces apoptotic cell death in cerebral cortex of neonatal rats. In addition, we clearly demonstrate a reduction in PP2A subunits by glutamate during brain development. These results showed that glutamate exposure leads to neuronal damage in the neonatal cerebral cortex through regulation of PP2A subunit B. Since PP2A exerts a biological function only when all three subunits are present, it is important to confirm the change in expression of all three subunits [12, 29, 30]. However, in this study, there is a limitation that only the change in the expression of PP2A subunit B was confirmed. Although PP2A subunit B is an important subunit that regulates the function of the PP2A holoenzyme, it is necessary to additionally confirm the expression changes of the remaining structure subunit A and catalytic subunit C. Thus, further study is needed to explain the detailed mechanism of glutamate-induced neuronal damage related with all PP2A subunits.

## Conclusions

This study demonstrated that glutamate exposure in neonatal brain cortex resulted in neuronal cell death through downregulation of PP2A subunit B. PP2A subunit B is an important factor in the brain development of newborns. Therefore, we can suggested that PP2A subunit B can be used as a therapeutic agent for brain cell damage caused by glutamate.

## Data Availability

The data that support the findings of this study are available on request from the corresponding author on reasonable request.

## Abbreviations

cDNA: Complement DNA
DAB: 3,3′-diaminobenzidine
DTT: Dithiothreitol
ECL: Enhanced chemiluminescence
IPG: Immobilized pH gradient
MALDI-TOF: Matrix-assisted laser desorption ionization- time of flight
NMDA: N-methyl-d-aspartate
NO: Nitric oxide
PBS: Phosphate-buffered saline
PCR: Polymerase chain reaction
PP2A: Protein phosphatase 2A
PVDF: Poly-vinylidene fluoride
SDS-PAGE: Sodium dodecyl sulfate-polyacrylamide gel electrophoresis
TBST: Tris-buffered saline containing 0.1% Tween-20
TdT: Terminal deoxynucleotidyl transferase
TUNEL: Terminal deoxynucleotidyl transferase dUTP nick end labeling
